# Recurrent bacteremia with a hypermucoviscous *Escherichia coli* isolated from a patient with perihilar cholangiocarcinoma: insights from a comprehensive genome-based analysis

**DOI:** 10.1186/s12941-022-00521-7

**Published:** 2022-06-24

**Authors:** Bernd Neumann, Norman Lippmann, Sebastian Wendt, Thomas Karlas, Christoph Lübbert, Guido Werner, Yvonne Pfeifer, Christopher F. Schuster

**Affiliations:** 1 Division Nosocomial Pathogens and Antibiotic Resistance, Department of Infectious Diseases, Robert Koch Institute, Wernigerode Branch, 38855 Wernigerode, Germany; 2 Institute for Hospital Hygiene, Medical Microbiology and Clinical Infectiology, Paracelsus Medical University, Nuremberg General Hospital, 90419 Nuremberg, Germany; 3 Interdisciplinary Centre for Infectious Diseases, Leipzig University Hospital, 04103 Leipzig, Germany; 4 Division of Infectious Diseases and Tropical Medicine, Department of Medicine II, Leipzig University Hospital, 04103 Leipzig, Germany; 5 Division of Gastroenterology, Department of Medicine II, Leipzig University Medical Center, 04103 Leipzig, Germany; 6 Present Address: Current Address: Center for Pandemic Vaccines and Therapeutics (ZEPAI), Paul-Ehrlich-Institute, 63225 Langen, Germany

**Keywords:** WGS, Oxford NanoPore MinION, Cholangiocarcinoma, Gallbladder, String-test, Virulence factors, Plasmid, Capsule

## Abstract

**Background:**

*Escherichia coli* (*E. coli*) is a common human pathogen, responsible for a broad spectrum of infections. Sites of infection can vary, but the hepato-biliary system is of particular concern due to the infection-associated formation of gallstones and the spread of pathogens from the bile ducts into the bloodstream.

**Case presentation:**

The presented case is striking, as the detected isolate showed a positive string test. This hypermucoviscous phenotype is atypical for *E. coli* and a particular feature of hypervirulent *Klebsiella pneumoniae* (*K. pneumoniae*) variants.

**Objectives:**

To provide new insights into the genomic background of an *E. coli* strain with an unusual hypermucoviscous phenotype using hybrid short- and long-read sequencing approaches.

**Results:**

Complete hybrid assemblies of the *E. coli* genome and plasmids were done and used for genome based typing. Isolate 537–20 was assigned to the multilocus sequence type ST88 and serotype O8:H4. The strain showed a close relationship to avian pathogenic strains. Analysis of the chromosome and plasmids revealed the presence of several virulence factors, such as the Conserved Virulence Plasmidic (CVP) region on plasmid 537-20_1, including several iron acquisition genes (*sitABCD, iroABCDEN, iucABCD, hbd*) and the *iutA* gene encoding the receptor of the siderophore aerobactin. The hypermucoviscous phenotype could be caused by encapsulation of putative *K. pneumoniae* origin.

**Conclusions:**

Hybrid sequencing enabled detailed genomic characterization of the hypermucoviscous *E. coli* strain, revealing virulence factors that have their putative origin in *K. pneumoniae*.

**Supplementary Information:**

The online version contains supplementary material available at 10.1186/s12941-022-00521-7.

## Background

*Escherichia coli* is a Gram-negative, rod shaped, facultative anaerobic bacterium of the *Enterobacteriaceae* family. The species is well known to be a frequent and numerous intestinal colonizer and pathogen of animals and humans, while also being ubiquitously present in the environment [1, 2]. *E. coli* strains are commonly categorized by their ability to cause specific intestinal or extraintestinal infections. Extraintestinal *E. coli* (ExPEC) have a well-described repertoire of virulence factors, and distinct clonal lineages are spread out globally [3–5]. Apart from these properties, in few cases atypical observations of hypermucoviscous (hmv) *E. coli* strains have been described in the literature [6–9]. Hypermucoviscosity (usual mucoid appearance on agar plates) is usually a characteristic of certain strain types of the *Klebsiella pneumoniae* (*K. pneumoniae*) species and is typically detected using the “string-test” [9]. A strain is considered positive, if it presents a mucoid string (> 5 mm) when touched with a glass rod or inoculation loop [10, 11]. It is typically described for clinical isolates that are associated with severe and invasive infections of otherwise healthy and immunocompetent, non-risk patients [12, 13]. This phenotype seems to be conditioned by several genetic components, including distinct capsule types and virulence genes (*iucA*, *iutA*, *rmpA*, *rmpA2*) [13–16]. The hmv phenotype decreases the immunological host defenses and enhances the bacterial survival rates [17, 18].

In the presented case, a string-positive *E. coli* strain was isolated from a patient with recurrent bacteremia, conjecturally causative colligated with the patient’s cholestatic cholangitis. The occurrence of *E. coli* in the biliary tract is well known, for example as a cause of gallstones [19, 20]. Intestinal bacteria such as *E. coli*, especially ExPEC, are able to invade the biliary tract during bile stasis, resulting in an acute infection [21, 22]. Furthermore, these severe infections are able to overcome the biliary system and thus allow *E. coli* to invade the bloodstream, leading to acute bacteremia [23, 24]. Furthermore, Søgaard and colleagues stated that gastrointestinal, hepatobiliary, and urinary tract cancer may debut with *E. coli* community-acquired bacteremia [25].

In this study, we aimed to analyze the genetic background of an *E. coli* strain displaying an hmv phenotype, using state-of-the-art genome analyses, including short- and long-read sequencing techniques.

### Importance

Description of an unusual hmv *E. coli* isolated from a patient suffering from biliary tract carcinoma and recurrent bacteremia.

## Methods

### Case presentation and bacterial isolation

A 71-year-old male German patient presented to the emergency department with acute cholangitis caused by a perihilar cholangiocarcinoma, also known as “Klatskin tumor” [26, 27], with hepatic and lymphogenic metastases. Comorbidities included chronic kidney insufficiency (KDIGO G2), type 2 diabetes mellitus and paroxysmal atrial fibrillation. At the time of admission to the hospital, the patient had fever (39.7 °C) and elevated inflammatory values (leukocytes: 16 × 10^9^/L, C-reactive protein: 88 mg/L, interleukin-6: 493 pg/mL). A few weeks earlier, the patient received piperacillin/tazobactam for similar clinical symptoms in another hospital.

As part of the extended routine diagnostics, a string-test positive *E. coli* isolate was identified in 2/2 peripherally obtained blood culture pairs and subjected to a detailed microbiological analysis.

The patient underwent endoscopic retrograde cholangiography (ERC) and biliary drainage was re-established by exchange of two bile duct plastic stents. In addition, an antibiotic therapy was immediately initiated with piperacillin/tazobactam. Later, the patient received cefotaxime and metronidazole and—due to a lack of clinical improvement—imipenem/cilastatine. Despite the treatment, the patient experienced several septic episodes during the following two months. Blood cultures were intermittently positive for the string-test positive *E. coli* isolate despite various antibiotic therapies. Finally, the patient received palliative chemotherapy and died 12 months after the initial diagnosis.

### In vitro characterization

*E. coli* 537-20 was isolated from patient blood cultures and identified as *Escherichia coli* via biochemical, phenotypical tests and matrix-assisted laser desorption ionization-time of flight mass spectrometry (MALDI-TOF). The isolate displayed an hmv phenotype on agar plates. A string test, typically used for hmv *K. pneumoniae*, was conducted to verify this phenotype. The string test was rated as positive as a mucoid string of > 5 mm could be observed, when touching bacterial plate growth with a standard inoculation loop and gentle pulling away, as described in the literature [11].

Antibiotic susceptibility testing (AST) was done using a VITEK 2 (GN AST N248) and a broth microdilution method, as described before [28]. The obtained results were interpreted according to EUCAST (European Committee on Antimicrobial Susceptibility Testing) standards and breakpoints v12.0 (https://eucast.org/clinical_breakpoints/). The following substances were used for AST: ampicillin, trimethoprim, cefotaxime, ceftazidime, gentamycin, chloramphenicol, colistin, nalidixic acid, ciprofloxacin, meropenem, sulfamethoxazole-trimethoprim, piperacillin, piperacillin-tazobactam, aztreonam, cefepime, tobramycin, amikacin and fosfomycin.

To investigate the general plasmid content and plasmid size, an S1-nuclease restriction and pulsed-field gel electrophoresis (PFGE) was performed, as described elsewhere [29]. The transferability of resistance genes by conjugative plasmids and a possible co-transfer of other phenotypic properties was investigated in a broth mating experiment using the sodium azide-resistant *E. coli* strain J53 Azi^r^ as recipient.

### Whole-genome sequencing, downstream data processing and assembly

DNA was extracted using the DNeasy Blood and Tissue Kit (Qiagen) and the MagAttract Kit (Qiagen) for high molecular weight DNA. The Qubit dsDNA HS Assay Kit (Invitrogen) was used for DNA quantification. DNeasy extracted DNA was sequenced on a NextSeq2000 benchtop device (Illumina) as described before [14]. The short-read whole-genome sequencing data analysis workflow was performed as described before, including several steps for quality control [14]. Long read sequencing was done similar as described before [30]. To this end high molecular weight DNA was size selected using SPRISelect beads (Beckman Coulter) and subjected to long-read sequencing with barcode 5 of the rapid barcoding kit (SQK-RBK004) on an R9.4 (FLO-MIN106) MinION flow cell and a Mk1c device for 21 h with live fast base-calling using guppy (v4.2.3) and auto de-multiplexing. This resulted in 137 k passed reads and 0.822 Gbp data for isolate 537–20. Reads were quality controlled using pycoqc (v2.5.0.23, https://github.com/a-slide/pycoQC) and kraken (v1.0) using an 8 GB mini kraken database. Adaptors were trimmed with porechop (v0.2.4, https://github.com/rrwick/Porechop) and the best 500 Mbp selected using filtlong (v0.2.0) and otherwise default parameters. Adaptor-clipped Illumina and filtered long-read data were hybrid assembled with Unicycler (v0.4.9b) [31] using default parameters, except for pinning SPAdes to version v3.13.0. The assembly was annotated with PGAP [32], first locally and later upon submission at and through NCBI again. One contig of 1760 bp was almost identical to a stretch of plasmid p537-20_1, had a reported depth of 0.4 × (chromosome was 1.04 ×), did not result in a circular contig and did not contain any plasmid replication genes. This contig was therefore removed from the assembly as it was thought to be an artifact.

### In silico characterization and prediction of virulence and resistance

The downstream analyses included the SeqSphere^+^ software suite (v7.7.5) [33] and web tools provided by the center for genomic epidemiology, including ResFinder (v4.1) [34], VirulenceFinder (v2.0) [35] and the Mobile Element Finder (v1.0) [36]. Also, the EnteroBase (v1.1.2) [37] platform was used for typing and investigations on a population level, including wgMLST SNP analyses and phylogenetic investigation of comparative isolates from Europe. Further, the Kleborate (v2.0.0) tool was used to investigate potential *Klebsiella*-related virulence traits [38]. The PLSDB was used to identify plasmid replicon types [39] and BLASTN was used to identify closely related plasmids. To this end, the whole plasmid sequence was used as a query in a BLASTN (megablast) [40] search against NCBI nr/nr and the results sorted according to accession length (ascending), percent identity (descending) and finally query cover (descending). The top three results were then selected. CGView Server BETA v0.1 [41] was used for visualization purposes of plasmids. Whole plasmid alignments were done with LASTZ (v1.02.00) [42, 43] (http://www.bx.psu.edu/~rsharris/lastz/) in the Geneious software (v2021.2.2, https://www.geneious.com) using either p537-20_1 or the comparison plasmid as a reference.

## Results and discussion

### Microbiological investigation of of *E. coli* strain 537–20

**Fig. 1:**
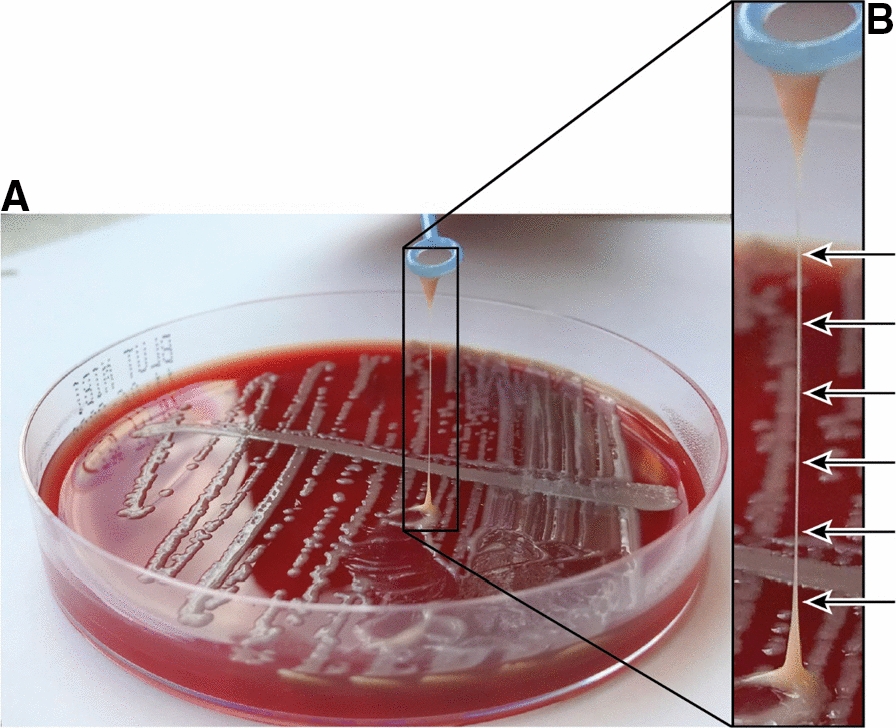
String test of *E. coli* isolate 537–20. **A** Strain was streaked out on Müller-Hinton sheep blood agar and incubated overnight at 37 °C. An inoculation loop (blue) was rubbed onto the colonies and pulled up vertically, forming a string (string test positive). **B** Close-up of string formed and indicated by arrows

Strain 537–20 was isolated from blood cultures of an elderly patient suffering from advanced perihilar cholangiocarcinoma and was identified as *Escherichia coli* via MALDI-TOF. AST revealed susceptibility of strain 537–20 to all tested antibiotics with exception of nalidixic acid and moxifloxacin. The in vitro* t*ransfer of these quinolone resistances in a broth mating experiment using the *E. coli* J53 recipient strain was not successful, incidicating a chromosomal, not plasmid based origin. Strain 537–20 showed a positive string test (Fig. 1), that has been typically described for *K. pneumoniae* strains and is caused by overproduction of mucus, an important feature of many hypervirulent *K. pneumoniae* strains [15]. The hypermucoviscosity characteristic in *K. pneumoniae* is discussed as general advantage for invasive infections, also considering the associated fitness costs [44] but the genetic cause in *E. coli* is unclear.

### Bacterial strain typing and chromosomal investigations

**Fig. 2:**
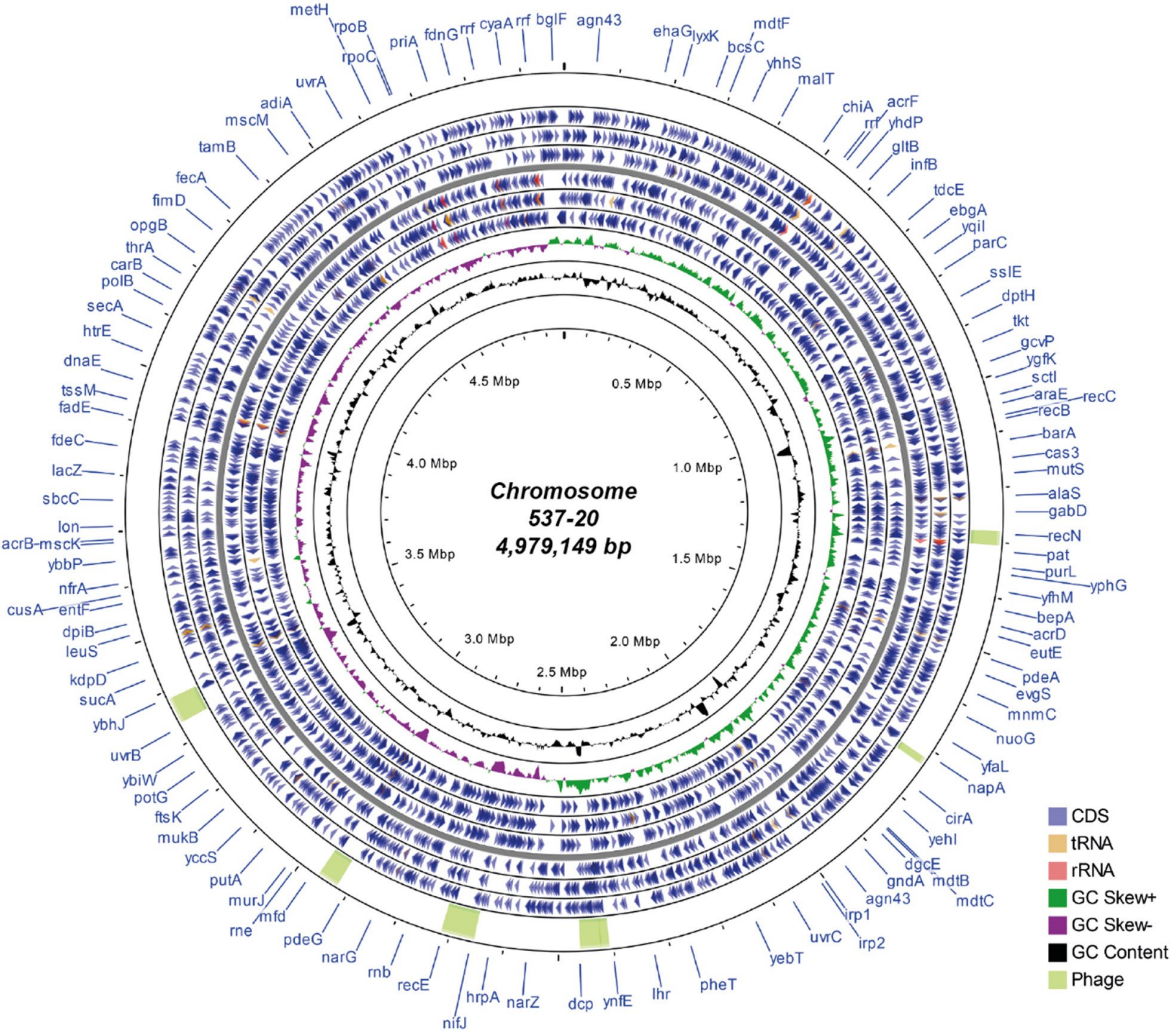
Chromosome of *E. coli* isolate 537–20 (CP091534). Coding sequences (CDS) are displayed separately for each of the six frames (rings with blue wedges). Prophages (as detected by Phaster [57, 58]), are shown in the outmost ring in green. tRNA: transfer RNA, rRNA: ribosomal RNA, GC skew ± : overabundance or lack of GC nucleotides. Image created with CGview Server v0.1 (http://cgview.ca/) [41]

The complete, circular chromosome assembly of isolate 537–20 was 4,979,149 bp long (Fig. 2, Additional file 1: Table S1) and was used for in silico typing using Enterobase (Table 1) [37]. The isolate was assigned to *Escherichia* phylogroup C, serotype O8:H4, multilocus sequence type ST88 (Achtmann scheme) and cgMLST type ST173013. The SeqSphere cgMLST type was CT12213.

**Table 1:** Results of in silico genotyping approaches

Typing scheme	Software/typing tool	Typing result
Phylogenetic group (Clermont type)	EnteroBase	C
Serotyping	SeroTypeFinder (CGE) / Enterobase	O8:H4
Multilocus sequence typing (MLST)	EnteroBase / SeqSphere +	ST88 (Achtmann scheme)
Core genome (cg)MLST	EnteroBase	ST173013
Core genome (cg)MLST	SeqSphere +	CT12213
Whole genome (wg)MLST	EnteroBase	ST196560
Capsule typing	Kleborate	KL54

*E. coli* of phylogenetic group C have been described as of commensal bacteria in humans and birds, but have been also reported in the clinical context [45, 46]. Serotype O8 has been commonly identified in clinical *E. coli*, but also in strains isolated from animals and sewage. [47–50]. Serotype O8 *E. coli* isolates are common EHEC variants; but the present isolate 537–20 did not contain a shiga toxin gene locus [48, 51]. The identified sequence type ST88, which is frequently associated with ExPEC strains in Europe, has been described as associated with colonization or urinary tract-infections, but not with bloodstream-infections [52]. Apart from that, a study by de Lastours et al*.* could show an association of increased mortality for bloodstream infections with the particular *E. coli* genotype combination ST88 and phylogenetic group C [53].

Analyzing the chromosomally encoded capsule of strain 537–20 using the *Klebsiella*-specific tool Kleborate resulted in the *Klebsiella*-capsule type KL54 (78.9% identity) and O5 (91.45% identity). The presence of *Klebsiella*-capsule genes in *E. coli* isolates seems to be uncommon, but not rare. Nanayakkara et al. [54] showed a wide diversity of *Klebsiella*-capsule types in *E. coli* isolates from Australia and were able to derive associations with *E. coli* subgroups. Their analyses revealed that the phylogenetic group C and serotype O8 are associated with emergence of *Klebsiella*-capsules. The detection of genes of a putative *Klebsiella*-capsule might be an explanation for the hmv phenotype of strain 537–20, especially since capsule types KL54have been found associated with positive string test results in previous studies [55]. Detailed functional studies are necessary to confirm the association between assessment of this capsule type and the hmv phenotype. Further, by applying the Kleborate tool, the operon genes for yersiniabactin and salmochelin were identified (Table2). This co-occurrence of the operons is a hint for the chromosomal integration of the *K. pneumoniae* integrative conjugative element (ICE*Kp*) [56]. Furthermore, we identified several *E. coli* virulence factors in the chromosome of strain 537–20, including genes encoding adherence proteins (*iha*), siderophores (*fyuA*) and iron transporters (*sitA*) among others (Table 2).

**Table 2:** Virulence and resistance features encoded on the 4,979,149 bp chromosome of strain 537–20 and plasmids p537-20_1 and p537-20_2

Chromosome 537–20 (CP091534)	Features
Virulence factors	*iha* (Adherence protein), *terC* (Tellurium ion resistance protein) [2x], *fyuA* (Siderophore receptor), *irp2* (High molecular weight protein 2 non-ribosomal peptide synthetase), *gad* (Glutamate decarboxylase) [2x], *sitA* (Iron transport protein), *iss* (Increased serum survival), *lpfA* (Long polar fimbriae), *mchB* (Microcin H47 part of colicin H), *mchC* (MchC protein), *mchF* (ABC transporter protein MchF), *hra* (Heat-resistant agglutinin) yersiniabactin operon (*ybtS, ybtX, ybtQ, ybtP, ybtA, irp2, irp1, ybtU, ybtT, ybtE, fyuA*) salmochelin operon (*iroB, iroC, iroD, iroN*)
Resistance features	*gyrA* (p.S83L) fluoroquinolone resistance
Plasmid p537-20_1 (CP091535)	
Virulence factors	*sitA* (Iron transport protein), *iucA* *iucB* *iucC* (Aerobactin synthetase), *iucD* *iutA* (Ferric aerobactin receptor), *etsC* (Putative type I secretion outer membrane protein) [2x], *iss* (Increased serum survival), *iroN* (Enterobactin siderophore receptor protein), *hlyF* (Hemolysin F), *mchF* (ABC transporter protein MchF), *cvaC* (Microcin C), *tsh* (Temperature-sensitive hemagglutinin), *ompT* (Outer membrane protease (protein protease 7)), *traT* (Outer membrane protein complement resistance)
Plasmid p537-20_2 (CP091536)	
Toxins	*cea* (Colicin E1) [2x]
Antibiotic resistances	Nalidixic acid, moxifloxacin

We further hypothesized, that the hmv phenotype of strain 537–20 could be caused by genome- or mobilome- integrated phages, that induce bacterial cell lysis. Hence we investigated the occurrence of phages in chromosome using PHASTER [57, 58]. We identified four intact phages (PHAGE_Entero_lambda_NC_001416, PHAGE_Escher_HK639_NC_016158, PHAGE_Entero_mEp460_NC_019716, PHAGE_Entero_DE3_NC_042057) from the Siphoviridae family on the chromosome with sizes of 39–62 kbp and three incomplete phages with sizes of 16–25 kbp. Some of the identified phages (e.g. the Lambda phage) can exhibit lytic life cycles that could lead to bacterial lysis. However, because these phages are also present in other, non-hmv *E. coli* strains, we have no evidence for an involvement of these phages in the observed hmv phenotype.

The genetic background of quinolone resistance of strain 537–20 was identified subsequently by analysis of the *gyrA* gene sequence. The detected 1 bp mutation in *gyrA* resulting in amino acid substitution S38L has been described to cause quinolone resistance [59, 60]. This was in accordance to the MIC results, which indicated nalidixic acid resistance and the mating experiments which pointed to a chromosomal source of resistance. Interestingly, this gyrase modification bas been found to be implicated in reduced virulence by reducing the expression of *fimA*, *papA*, *papB* and the *ompA* genes resulting in decreased capacity to cause cystitis and pyelonephritis [61].

### Plasmid analyses

**Fig. 3:**
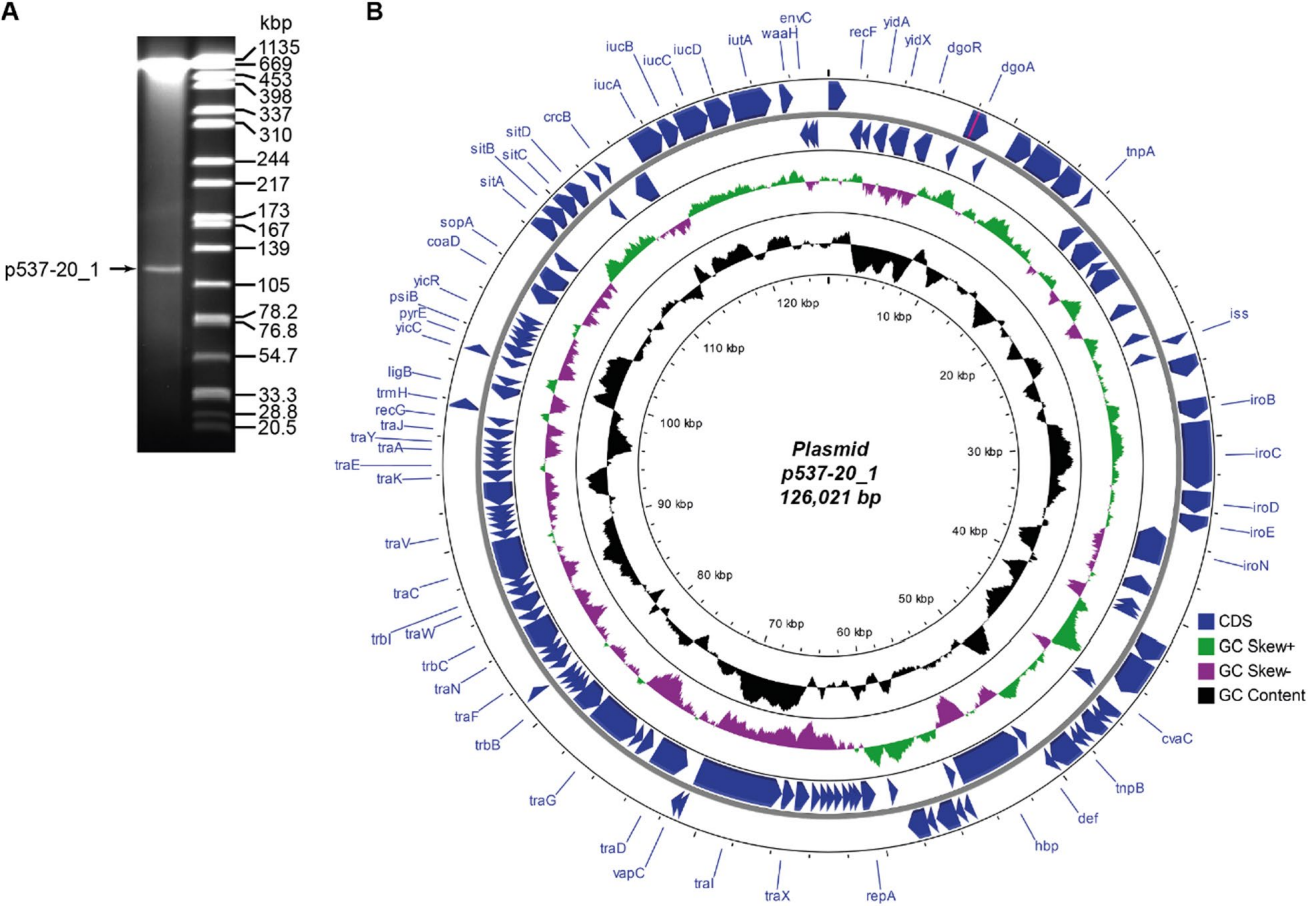
Analysis of plasmid p537-20_1 (CP091535). **A** Visible plasmids after S1 nuclease restriction and PFGE (right lane, molecular marker strain *S.* Braenderup H9812, restricted with XbaI). **B** Map of plasmid p537-20_1. Image created with CGview Server v0.1 (http://cgview.ca/) [41]

S1-PFGE analysis indicated the presence of at least one large plasmid of approx. 120 kbp in isolate 537–20 (Fig. 3a). Several smaller plasmids of approximately 6000 bp, 2100 bp and 1500 bp (double band) were visible in a native plasmid preparation (Qiagen plasmid mini Kit) (Additional file 1: Fig. S1).

In accordance, five plasmids were present in the final short and long-read hybrid assembly (Table 3): one large plasmid of 126,021 bp (p537-20_1) (Fig. 3b) and four smaller plasmids of 6,647 bp (p537-20_2), 2,182 bp (p537-20_3), 1,552 bp (p537-20_4) and 1,433 bp (p537-20_5).

**Table 3:** Plasmids present in isolate 537–20

Plasmids	Accession	Size [bp]	Closest Relative(s) based on BLASTN	BLASTN (coverage/identity)	Plasmid replicon type
p537-20_1	CP091535	126,021	pPK8261-180 kb (180,318 bp) pWP5-W18-ESBL-11_1 (158,291 bp) pWP4-S18-ESBL-08_1 (158,292 bp)	CP080157.1 (98/99.79%) AP022121.1 (97/99.88%) AP022096.1 (97/99.88%)	0.99952 identity IncFIC(FII)_1, AP001918 IncFIB(AP001918)_1, AP001918
p537-20_2	CP091536	6,647	pRHB38-C04_2 (6,647 bp) p94EC-5 (6,647 bp) RCS33_pII (6,647 bp)	CP057102.1 (100/100%) CP047581.1 (100/100%) LT985237.1 (100/100%)	1.0000 Identity ColRNAI_1, DQ298019
p537-20_3	CP091537	2,182	pRHBSTW-00087_3 (2,181 bp) p5312.29 (86,455 bp) pWP4-S18-ESBL-09_5 (2,188 bp)	CP056869.1 (100/99.95%) KR905385.1 (82/98.55%) AP022103.1 (63/92.63%)	n.a
p537-20_4	CP091538	1,552	p7_2.5 (1,552 bp) pET6.4-ColRNAI (1,552 bp) pSCU-172–9 (1,552 bp)	CP023825.1 (100/100%) CP043221.1 (100/99.94%) CP054362.1 (100/99.92%)	0.99933 identity Col(MG828)_1, NC_008486
p537-20_5	CP091539	1,433	MINF_9A-sc-2280436, plasmid 9 (1,433 bp) Uncultured metamobilome (1,454 bp) E. coli M718 genome assembly (5,444,046 bp)	LR890342.1 (100/100%) LN852838.1 (100/100%) OU349840.1 (86%/94.34%)	0.999281 identity Col(MG828)_1, NC_008486

Closest relatives were identified using the NCBI BLASTN suite and the megablast algorithm against the nr/nt database (Jan 16th 2022)

The hits shown were selected by sorting the best 100 hits by first accession length (ascending), then percent identity (descending) and finally query cover (descending)

The top three hits were selected. Plasmid replicon type was determined using PLSDB [39]

The plasmid p537-20_1 was of IncFIC(FII)_1/IncFIB(AP001918)_type, carried a *vapBC* toxin-antitoxin system, a potential *hok/sok* toxin-antitoxin system, several virulence genes, including the increased serum survival gene *iss* and, regulatory genes for iron metabolism (*iroBCDEN*, *iucABCD*, *sitABCD, hbp* [hemoglobin-binding protease autotransporter Hbp]). Many of these virulence genes and loci belong to the previously described Conserved Virulence Plasmidic (CVP) region. Lemaître et al*.* proposed the CVP region to be responsible for the virulence of an ExPEC strain of *E. coli* phylogroup C [46]. A LASTZ alignment using the original CVP sequence (HF922624) [46] as a reference, revealed that plasmid 537-20_1 contained most features of the CVP (Additional file 1: Fig. S2) with the main difference being an inversion of the *sitABCDE-iucABCD-iutA* region. When p537-20_1 served as the reference, the absence of a 40 kbp *tra* region was noted, but this could be attributed to sequence HF922624 possibly not including the full plasmid but only the CVP region (Additional file 1: Fig. S2). We further compared p537-20_1 to the sequence of the CVP containing plasmid pECOS88 (CU928146), which was proposed to be associated with meningitis in neonates and displaying high levels of bacteremia in a neonatal rat model [62]. The two plasmids showed a high degree of similarity in structure (Additional file 1: Fig. S3). Of note, p537-20_1 also contained the *hbp* gene which was absent in pECOS88. *Hbp* is a protease that is involved in host hemoglobin proteolysis and used to acquire iron from the host [63]. The *hbp* gene was shown to be associated with iron-limited infection-sites (e.g. intra-abdominal abscesses, [63] which might have served as a source of the blood stream infection). Interestingly, p537-20_1 also carried the *iutA* gene encoding the receptor of aerobactin, which is an important virulence factor for ExPEC and hypervirulent *K. pneumoniae* [52]. The carriage of *iutA* has been shown to be associated with increased mortality in *E. coli*, as well as *K. pneumoniae* bloodstream infection [23, 64]. The possession of the *iutA* gene but also many other iron acquisition genes likely represents a fitness advantage in the biliary tract, due to the general iron limitation in bile [23, 65]. The co-occurrence of *iutA* and *iucA* in APEC *E. coli* was described before, but as characteristic of Col (V) plasmids [66].

The other plasmids of strain 537-20 were considerably smaller and did not contain any notable features, except for two *cea* (Colicin E1) genes in p537-20_2. Plasmid p537-20_2 was identified as a ColRNAI plasmid and plasmids p537-20_4 and p537-20_5 as Col(MG828) plasmids. No replicon information could be identified for plasmid p537-20_3.

### Global phylogenetic comparison

**Fig. 4:**
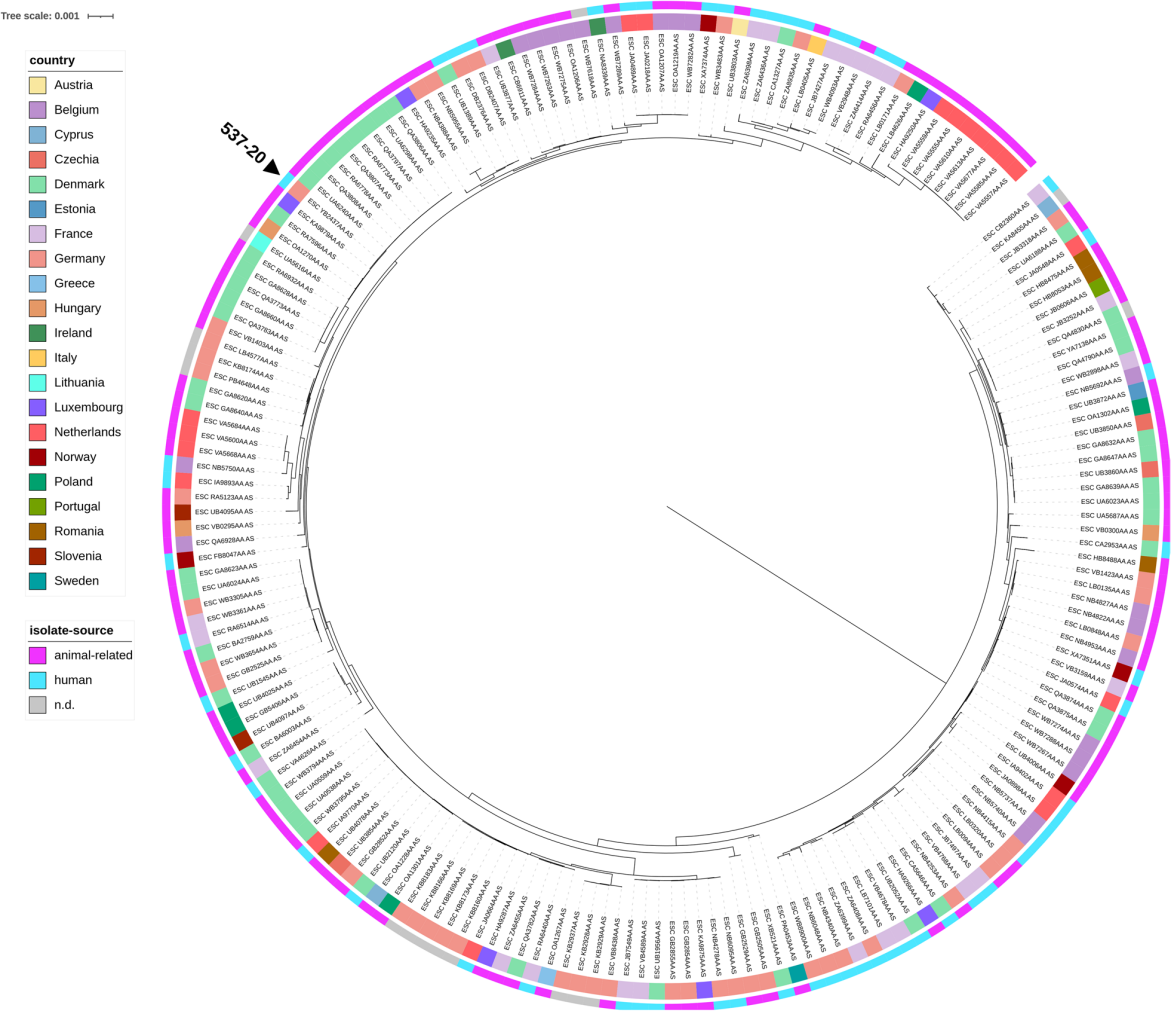
Phylogenetic tree based on wgMLST SNP analyses of 194 European ST88 isolates, submitted to EnteroBase, and strain 537–20. A phylogenetic based on wgMLST SNP analyses of European ST88 isolates, submitted to EnteroBase. Visualization was realized with iTOL (v6.3) and information about isolate origin (country and isolation source) were added

We further investigated the relationship of strain 537–20 to other *E. coli* isolates of the same sequence type. A total of 194 *E. coli*-ST88 isolates submitted to EnteroBase, were subjected for wgMLST SNP analyses. These originated from 21 European countries, including human origin and animal-associated origins (animals, livestock, food). The resulting phylogenetic tree (Fig. 4) visualizes the population structure of ST88 isolates.

The data analysis showed that *E. coli* ST88 is widely distributed in both human and animal-associated resources. Generally, ST88 is common for the European region [52]. Human- associated clusters can be observed, as well as animal-associated clusters. Other ST88 isolates of human origin, especially from Germany, were clearly separated from strain 537–20 in the phylogenetic tree (Fig. 4). Surprisingly, strain 537–20 clustered closely with isolates of animal origin (poultry/livestock) from Luxembourg and Denmark. This finding supports a hypothesis of a putative zoonotic origin, also because CVP containing plasmids were shown to be linked to extraintestinal avian pathogenic *E. coli* (APEC) [46, 62].

## Conclusions

The hybrid sequencing approach allowed deep insights in the genome and plasmidome of the hypermucoviscous *E. coli* strain 537–20 causing recurrent bacteremia. The cause of the hypermucoviscous phenotype remains speculative, it might be due to the expression of a capsule of putative *K. pneumoniae* origin. As has been discussed for uropathogenic hypermucoviscous *E. coli* isolates [8], the direct linkage between the hmv phenotype and the clinical outcome is difficult to determine and raises the question of routine string-test screening.

The conducted typing and comparative phylogenetic analyses revealed a close relationship of this ST88 strain to ExPEC and APEC isolates. The virulence potential could be traced back to the acquisition of a conserved plasmid-located virulence island, the CVP region and ICE*Kp*, that is a common virulence mediating element in *K. pneumoniae*. In addition, plasmid p537-20_1 contains several iron acquisition genes that enable growth under iron limiting conditions such as in the bile. Further in-depth studies are needed to investigate the interactions between this *E. coli* strain and human host its role in the processes of infection in bile duct and blood.

## Supplementary Information

**Additional file 1: Figure S1.** Visualization of plasmids of strain 537-20 by native plasmid preparation (Plasmid Mini Kit, Qiagen, Hilden, Germany) and agarose gel electrophoresis. The plasmid containing *E. coli* strain V515 was used as a reference (lane M). Several plasmids were visible in strain 537-20 (lane 1).Plasmid sizes that were bioinformatically identified are indicated on the right. **Figure S2.** LASTZ alignment of p537-20_1 (CP091535) and Conserved Virulence Plasmidic (CVP) region (HF922624). In the top panel, plasmid p537-20_1 was aligned to the CVP region (HF922624, reference) using LASTZ. The LASTZ algorithm allows to identify regions of similarity as indicated in the “LASTZ Alignment Graph". Blue regions indicate identity, whereas red regions indicate inversions compared to the reference sequence. The X-axis in the graph describes the bp location. In the lower panel, reference and comparison sequences are switched to identify regions that are absent in the reference sequence. **Figure S3.** LASTZ alignment of p537-20_1 (CP091535) and pECOS88 (CU928146). **Table S1.** Sequencing and assembly statistics.

## Data Availability

Raw read data of both, Illumina and MinION were submitted to NCBI and are available under BioProject accession PRJNA800416, BioSample accession SAMN25247371 and Sequence Read Archive (SRA) accession numbers SRR17758648 (Illumina) and SRR17758649 (MinION). Hybrid assembly is available under accession numbers CP091534-CP091539 and the short-read assembly is available in EnteroBase (Uberstrain ESC_WA1159AA).

## Abbreviations

APEC: Extraintestinal avian pathogenic *E. coli*
AST: Antibiotic susceptibility testing
CVP: Conserved virulence plasmidic region
ERC: Endoscopic retrograde cholangiography
ExPEC: Extraintestinal *E. coli*
hmv: Hypermucoviscous
MALDI-TOF: Matrix-assisted laser desorption ionization-time of flight mass spectrometry
PFGE: Pulsed-field gel electrophoresis
