# Supplementary material for: Recurrent bacteremia with a hypermucoviscous Escherichia coli isolated from a patient with perihilar cholangiocarcinoma: insights from a comprehensive genome-based analysis
Source: Ann Clin Microbiol Antimicrob. 2022 Jun 24;21:28. doi: 10.1186/s12941-022-00521-7 (PMC9233328; doi:10.1186/s12941-022-00521-7)
Supplement: Supplementary file 1 — Additional file 1: Figure S1. Visualization of plasmids of strain 537-20 by native plasmid preparation (Plasmid Mini Kit, Qiagen, Hilden, Germany) and agarose gel electrophoresis. The plasmid containing E. coli strain V515 was used as a reference (lane M). Several plasmids were visible in strain 537-20 (lane 1).Plasmid sizes that were bioinformatically identified are indicated on the right. Figure S2. LASTZ alignment of p537-20_1 (CP091535) and Conserved Virulence Plasmidic (CVP) region (HF922624). In the top panel, plasmid p537-20_1 was aligned to the CVP region (HF922624, reference) using LASTZ. The LASTZ algorithm allows to identify regions of similarity as indicated in the “LASTZ Alignment Graph". Blue regions indicate identity, whereas red regions indicate inversions compared to the reference sequence. The X-axis in the graph describes the bp location. In the lower panel, reference and comparison sequences are switched to identify regions that are absent in the reference sequence. Figure S3. LASTZ alignment of p537-20_1 (CP091535) and pECOS88 (CU928146). Table S1. Sequencing and assembly statistics. [file 12941_2022_521_MOESM1_ESM.docx]

**SUPPLEMENTARY INFORMATION
FOR**

Brief report - Annals of Clinical Microbiology and Antimicrobials

**Recurrent bacteremia with a hypermucoviscous *Escherichia coli* isolated from a patient with perihilar cholangiocarcinoma: insights from a comprehensive genome-based analysis.**

Bernd Neumann^1,2^*, Norman Lippmann^3^, Sebastian Wendt^3,4^, Thomas Karlas^5^, Christoph Lübbert^3,4^, Guido Werner^1^, Yvonne Pfeifer^1^, Christopher F. Schuster^1,6^*

^1^ Division Nosocomial Pathogens and Antibiotic Resistance, Department of Infectious Diseases, Robert Koch Institute, Wernigerode Branch, 38855 Wernigerode, Germany

^2^ Institute for Hospital Hygiene, Medical Microbiology and Clinical Infectiology, Paracelsus Medical University, Klinikum Nürnberg, 90419 Nuremberg, Germany

^3^ Interdisciplinary Centre for Infectious Diseases, Leipzig University Hospital, 04103 Leipzig, Germany

^4^ Division of Infectious Diseases and Tropical Medicine, Department of Medicine II, Leipzig University Hospital, 04103 Leipzig, Germany

^5^ Division of Gastroenterology, Department of Medicine II, Leipzig University Medical Center, 04103 Leipzig, Germany

^6^ Current address: Center for Pandemic Vaccines and Therapeutics (ZEPAI), Paul-Erlich-Institute, 63225 Langen, Germany

*corresponding authors


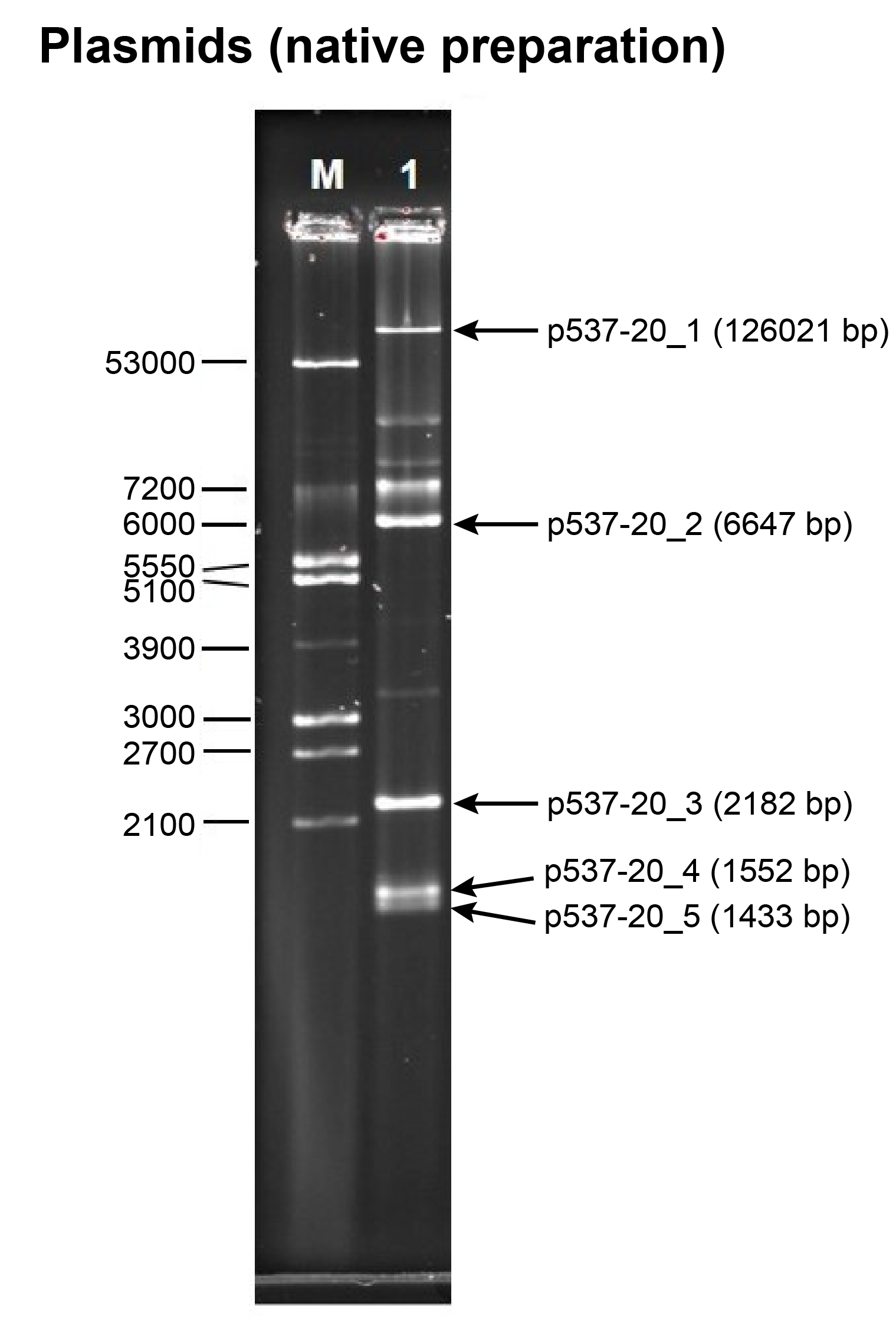


**Figure S1: Visualization of plasmids of strain 537-20 by native plasmid preparation** ( Plasmid Mini Kit, Qiagen, Hilden, Germany) **and agarose gel electrophoresis.** The plasmid containing *E. coli* strain V515 was used as a reference (lane M). Several plasmids were visible in strain 537-20 (lane 1).Plasmid sizes that were bioinformatically identified are indicated on the right.


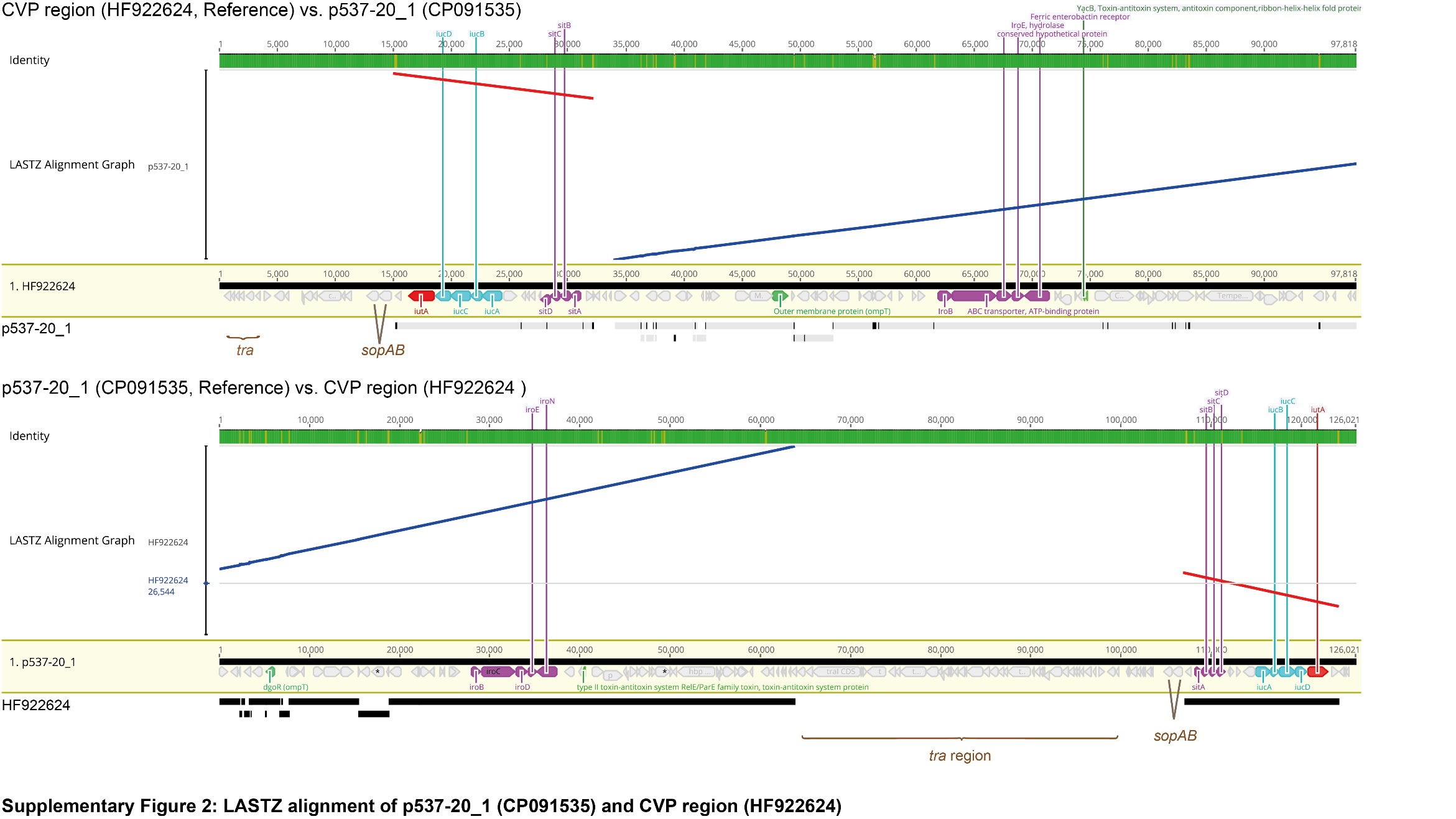


**Figure S2: LASTZ alignment of p537-20_1 (CP091535) and** Conserved Virulence Plasmidic (**CVP) region (HF922624).** In the top panel, plasmid p537-20_1 was aligned to the CVP region (HF922624, reference) using LASTZ. The LASTZ algorithm allows to identify regions of similarity as indicated in the “LASTZ Alignment Graph". Blue regions indicate identity, whereas red regions indicate inversions compared to the reference sequence. The X-axis in the graph describes the bp location. In the lower panel, reference and comparison sequences are switched to identify regions that are absent in the reference sequence.

Plasmid p537_20_1 and the CVP region share two regions containing *sitABCD* & *iucABCD* and *ompT* & *iroBCDEN* of which one is in opposite orientation*.* The transfer (*tra*) region and partitioning systems (*sopAB*) do not match between the two plasmids in either alignment, indicating important differences between the two plasmids.

**
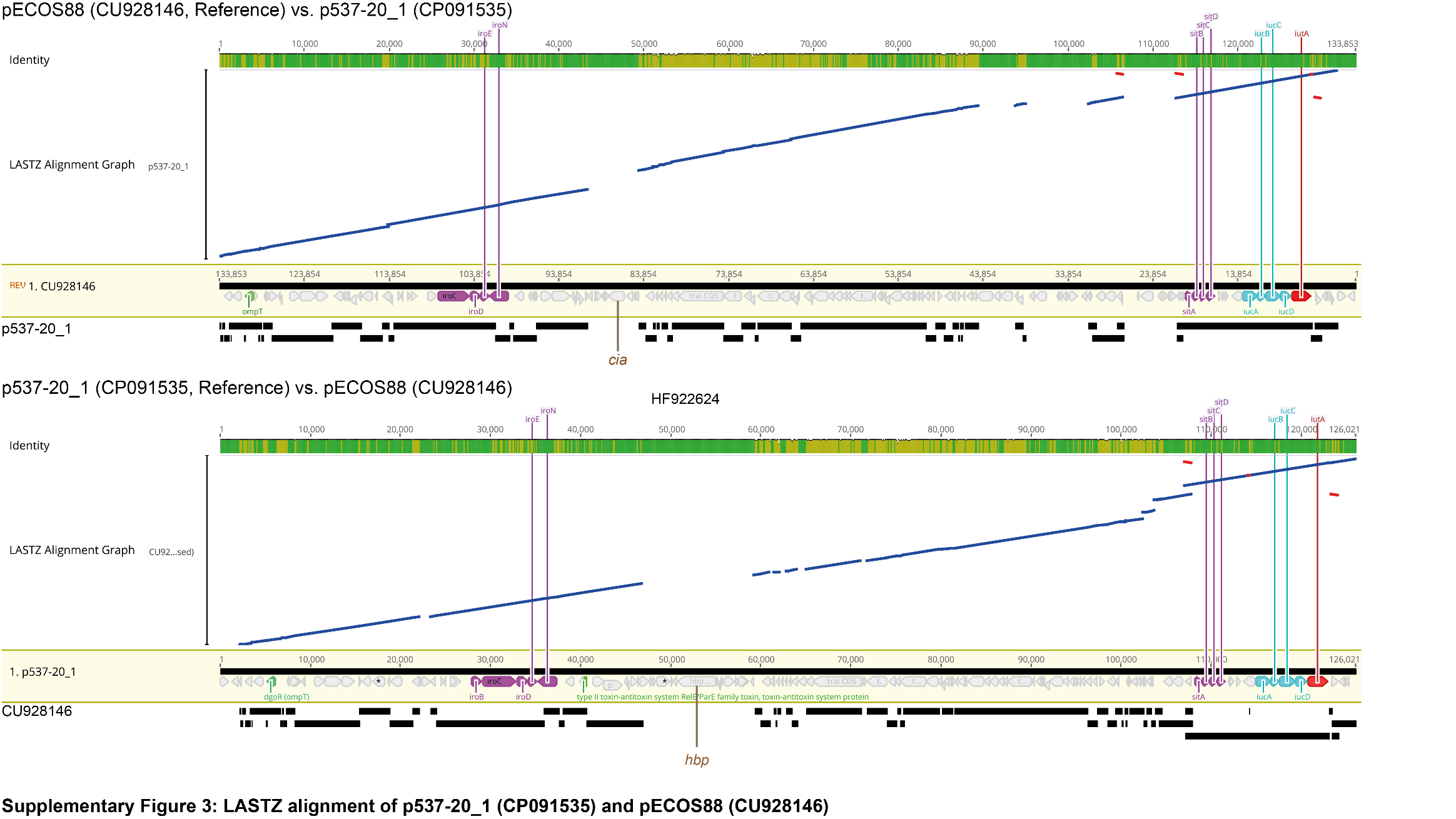
**

**Figure 3: LASTZ alignment of p537-20_1 (CP091535) and** **pECOS88 (CU928146).**

In the top panel, plasmid p537-20_1 was aligned to plasmid pECOS88 (CU928146, reference) using LASTZ. The LASTZ algorithm allows to identify regions of similarity as indicated in the “LASTZ Alignment Graph". Blue regions indicate identity between the sequences and the X-axis describes the bp location. In the lower panel, reference and comparison sequences are switched to identify regions that are absent in the reference sequence.

Plasmid p537_20_1 and plasmid pECOS88 share large regions with each other. Interestingly, p537_20_1 contained the *hbp* gene (involved in host hemoglobin proteolysis) which was missing in plasmid pECOS88.

**Table S1: Sequencing and assembly statistics:**

| **Parameter** | **Finding** |
| --- | --- |
| **Species** | *Escherichia coli* |
| **Strain** | 537-20 |
| **Year of isolation** | 2020 |
| **Location** | Germany |
| **Host** | Human (*Homo sapiens*) |
| **Isolation source** | Blood culture |
|  |  |
| **Illumina sequencing (paired)** |  |
| Number of reads | 4,305,954 |
| Throughput (bp) | 626,739,762 |
| Average coverage (×) | 122 |
| SRA accession number | [SRR17758648](https://www.ncbi.nlm.nih.gov/sra/SRR17758648) |
| **MinION sequencing** |  |
| Number of reads | 137,867 |
| Throughput (bp) | 822,132,215 |
| Median read length | 2,587 |
| Median read quality | 11.0 |
| Read length N50 (bp) | 13,212 |
| Average coverage (×) | 161 |
| SRA accession number | [SRR177586489](https://www.ncbi.nlm.nih.gov/sra/SRR17758649) |
|  |  |
| **Hybrid Assembly** |  |
| Number of contigs | 6 |
| Total genome size (bp) | 5,118,744 |
| Chromosome size (bp) | 4,979,149 |
| Annotation Pipeline | NCBI Prokaryotic Genome Annotation Pipeline (PGAP) |
| Annotation Method | Best-placed reference protein set; GeneMarkS-2+ |
| Annotation Software revision | 5.3 |
| Features Annotated | Gene; CDS; rRNA; tRNA; ncRNA; repeat_region |
| Genes (total) | 4,939 |
| CDSs (total) | 4,816 |
| Genes (coding) | 4,606 |
| CDSs (with protein) | 4,606 |
| Genes (RNA) | 123 |
| rRNAs | 8, 7, 7 (5S, 16S, 23S) |
| complete rRNAs | 8, 7, 7 (5S, 16S, 23S) |
| tRNAs | 90 |
| ncRNAs | 11 |
| Pseudo Genes (total) | 210 |
| CDSs (without protein) | 210 |
| Pseudo Genes (ambiguous residues) | 0 of 210 |
| Pseudo Genes (frameshifted) | 110 of 210 |
| Pseudo Genes (incomplete) | 111 of 210 |
| Pseudo Genes (internal stop) | 43 of 210 |
| Pseudo Genes (multiple problems) | 46 of 210 |
| CRISPR Arrays | 1 |

**Table S1 (continued).**

| **Assembly accession numbers** |  |
| --- | --- |
| 537-20 chromosome | [CP091534](https://www.ncbi.nlm.nih.gov/nuccore/CP091534) |
| p537-20_1 | [CP091535](https://www.ncbi.nlm.nih.gov/nuccore/CP091535) |
| p537-20_2 | [CP091536](https://www.ncbi.nlm.nih.gov/nuccore/CP091536) |
| p537-20_3 | [CP091537](https://www.ncbi.nlm.nih.gov/nuccore/CP091537) |
| p537-20_4 | [CP091538](https://www.ncbi.nlm.nih.gov/nuccore/CP091538) |
| p537-20_5 | [CP091539](https://www.ncbi.nlm.nih.gov/nuccore/CP091539) |
| BioSample accession number | [SAMN25247371](https://www.ncbi.nlm.nih.gov/biosample/SAMN25247371/) |
| BioProject accession number | [PRJNA800416](https://www.ncbi.nlm.nih.gov/bioproject/PRJNA800416) |
